# Passage Number of 4T1 Cells Influences the Development of Tumour and the Progression of Metastasis in 4T1 Orthotopic Mice

**DOI:** 10.21315/mjms2022.29.3.4

**Published:** 2022-06-28

**Authors:** Harishini Rajaratinam, Nur Syahmina Rasudin, Sabreena Safuan, Nurul Asma Abdullah, Noor Fatmawati Mokhtar, Wan Ezumi Mohd Fuad

**Affiliations:** 1 School of Health Sciences, Universiti Sains Malaysia, Kelantan, Malaysia; 2 Institute for Research in Molecular Medicine (INFORMM), Universiti Sains Malaysia, Kelantan, Malaysia

**Keywords:** cell line, breast cancer, metastasis, mice model, passage

## Abstract

**Background:**

The study was aimed to elucidate the influence of passage number of 4T1 cells for the development of the ideal tumour model.

**Methods:**

A total of 24 female BALB/c mice was divided equally into three groups: i) control (phosphate buffered saline [PBS] only); ii) group A (subjected to 4T1 cells of passage number 9) and iii) group B (subjected to 4T1 cells of passage number 10). The injections were introduced at the 3rd mammary pad of the mice. The net volume of the tumours was examined. Histopathological analysis was conducted to compare the extent of metastasis in the different groups of mice.

**Results:**

Group B had a higher net volume of 4T1 tumour as compared to group A (*P* = 0.042). The coefficient of variation in the net volume of 4T1 tumour for group A was higher (135.3%) as compared to group B (40.79%). Group A only exhibited metastasis on the lungs, liver and spleen whereas group B showed metastasis to the heart, spleen, lungs and liver.

**Conclusion:**

The use of 4T1 cells from passage number 10 is more ideal for the development of 4T1 tumour.

## Introduction

The 4T1 mice model is a well-known model that has been widely used in research to study breast cancer (1–4). The 4T1 tumour is commonly known for its highly tumourigenic nature and its capability to invade and spontaneously metastasise from the primary tumour in the mammary gland to multiple distant sites including lymph nodes, blood circulation and target organs such as the liver, lungs and brain (5–7). There are several characteristics of the 4T1 tumour that makes it an ideal model to mimic the condition of human mammary carcinoma, especially Stage IV triple-negative breast cancer (8). The 4T1 cells are easily transplanted and grow at the desired anatomical site. The progressive metastasis of 4T1 cells from the primary tumour to the draining lymph nodes and target organs mimics the same circumstances portrayed in human mammary cancer (5).

Passage number is the number of times, in which a cell culture has been sub-cultured and determining the ideal passage number can make or break an experiment. In the current study, we have attempted to develop a 4T1 orthotopic mice model by introducing 4T1 cells at the 3rd mammary fat pad of female BALB/c mice. The detailed protocol published by Pulaski and Ostrand-Rosenberg (1) was adopted in the current study. According to the protocol, cultures should be split 2–3 times per week. The study suggested that cultured cells should not be allowed to exceed growth confluency (ideally around 50%–80% of confluency) as the overgrowth of cells may affect cell viability (2). However, the protocol did not specifically report the passage number that was used in their protocol.

According to a study by Hoon, Wang and Ramshaw (9), passaging could lead to changes in the chromosomal stability in the cells of later passage which influences its metastatic capacity when introduced into animal models. The role of passage in influencing the nature of tumour growth in animal models has been highlighted by Bleijs et al. (10). It was suggested that early passages of patient-derived tumour xenografts (PDTXs) should be used for translational applications to prevent outcomes that can deviate from the clinical response. According to Morgan and colleagues (11), from the total number of mutations detected in primary non-small-cell-lung cancer tumours, only 43% were detected in the corresponding PDTXs and four additional mutations arose in early passages of PDTXs that were not present in the primary tumour.

Since passage number could play a role in modifying the biological profile of the culture cell, we have attempted to incorporate a similar idea in the development of 4T1 orthotopic mice. In the present study, we aimed to highlight the influence of the passage number of 4T1 cells on the volume of the 4T1 tumour bore by the host throughout the duration of tumour development and the pattern of tumour growth. Additionally, we would also like to compare the difference in the volume of 4T1 tumour between different passage numbers at five-week points of tumour development. The variation in the net volume of the 4T1 tumour due to the difference in passage numbers and the extent of metastasis within the hosts, were studied as well.

## Methods

### Animal Species, Supply and Ethics

A total of 24 female BALB/c mice aged around 5–6 weeks old were used to develop the 4T1 tumour. There were three groups (*n* = 8 in each group) of mice in this study: i) control mice group; ii) group A (subjected to passage number 9: hereafter referred to as P9) and iii) group B (subjected to passage number 10: hereafter referred to as P10). The animal study was conducted at the Animal Research and Service Centre (ARASC), Universiti Sains Malaysia (USM), Health Campus, Kelantan, Malaysia. The ethical approval for the animal study was granted by the Institutional Animal Care and Use Committee (IACUC), USM.

### Cell Culture

The 4T1 cells were purchased from the American Type Culture Collection (ATCC) and cultured using complete DMEM media (Gibco), supplemented with 10% foetal bovine serum (FBS) (Gibco) and 1% antibiotics (penicillin and streptomycin) (Gibco). For groups A and B, cells from P9 and P10 were harvested using 0.25% Trypsin (Gibco), respectively. For P9 cells, the stock cells were retrieved from the cryovial which was originally from P7 whereas for P10 cells, the stock cells were retrieved from the cryovial that was originally from P8. Therefore, the sub-cultivation process was only performed once for both batches of cells regardless of their passage number.

### Introduction of 4T1 Orthotopic Injection

Irrespective of the assigned passage number, the procedure for the preparation of 4T1 orthotopic injection was the same for both groups A and B. Approximately, 200 μL of 1×10^5^ 4T1 cells were isolated and introduced subcutaneously at the 3rd mammary fat pad of the BALB/c mice, in groups A and B. For the control mice, 200 μL of PBS was introduced at a similar site.

### Development of 4T1 Orthotopic Mice Model

The volumes of the 4T1 tumours were monitored over the period of 30 days for group A and 42 days for group B. The diameter and width of each tumour were measured and the final volume was calculated by using the formula: 0.52 × width^2^ × diameter (12). After 30 days, group A mice were euthanised via decapitation, after anesthetised with sodium pentobarbital. The euthanisation of group B and control mice was carried out after the completion of 42 days.

### Collection of Organs

The resected tumours and target organs such as the heart, liver, lungs and spleen were preserved in 37% formalin (Merck).

### Histopathology Analysis

Standard haematoxylin and eosin staining was used on paraffin-embedded target organs for histological examination of metastases. Stained sections were examined for signs of metastasis and photographed using Olympus BX41 light microscope and CellSensor software, respectively.

### Statistical Analysis

Graphpad Prism 8 software was used to generate the statistical analyses. The error bars either represent standard deviation (SD) or interquartile range (IQR). For the data that consisted of more than two groups with repeated measures, the Friedman test and its post-hoc analysis, Wilcoxon signed-rank test with Bonferroni’s corrections were carried out. For the analysis that involved only two groups, independent *t*-test was conducted for parametric analyses and the Mann-Whitney U test was conducted for non-parametric analyses.

## Results

### Physical and Behavioural Changes in the 4T1 Tumour Bearing Mice

**Figure 1:**
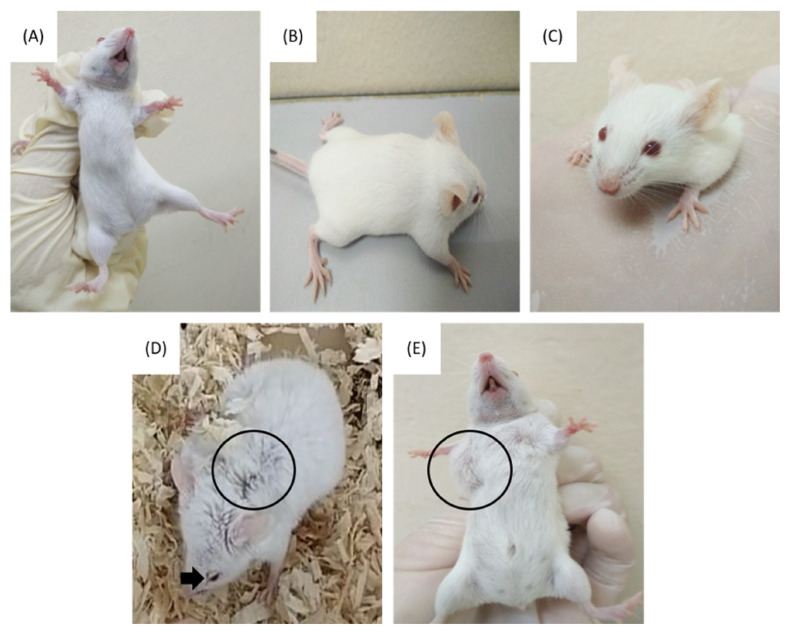
The panel shows the physical difference between control and 4T1 orthotopic mice.

In both groups A and B, palpable tumour generally appeared after 14 days of the introduction of the 4T1 orthotopic injection. In group A, palpable tumour was only present in six out of eight mice with varying sizes of tumour observed. Based on the physical appearance, only two mice displayed a larger size of tumour compared to the other four mice. Some prominent physical and behavioural changes were noted in these two mice which included pulled ears, rapid breathing, inactiveness and changes in the fur pattern from smooth to coarse (Figure 1). However, the other four mice that carried smaller sizes of tumour were much more active and hardly displayed the aforementioned symptoms, which was common among the two mice with larger tumour sizes. The other two mice that did not develop any palpable tumour were healthy and showed normal physical appearance and behaviour similar to those observed in their control counterparts. Meanwhile, the two mice which developed the largest tumour size showed drastic physical changes and signs of dying by the mid of week 5 (30 days) (Figure 1). This observation was earlier than expected, forcing the study to be stalled and all eight mice assigned under group A were sacrificed.

The upper panel **(A)**–**(C**) represents the normal physical appearance of the control mice: **(A)** The absence of palpable 4T1 tumour at the 3rd mammary fat pad of the control mouse. **(B)** The fur-coat of the control mouse was smooth till the end of the experimental duration. **(C)** The lids of the eyes of the control mouse did not appear as droopy. The lower panel **(D)**–**(E)** represents the physical appearance of the 4T1 orthotopic mice: **(D)** The fur-coat of the 4T1 orthotopic mouse seemed coarse (marked by the black circle) and the eyes lids were droopy (marked by the black arrow) at week 5. **(E)** There was a palpable tumour on the 3rd mammary fat pad of the 4T1 orthotopic mouse at week 5 (marked by the black circle)

In group B, the development of palpable tumour was successful in all eight mice with less variation in the size of tumour observed. Based on the physical appearance, by week 4 (day 22–28), all the mice showed extreme changes in their physical appearance. Among the changes noted were changes in the fur pattern, pulled ears, droopy eyes, breathing difficulties and reduced mobility due to the restriction caused by the growing 4T1 tumour (Figure 1). However, in week 5 (day 28–35), the size of the tumour obviously reduced in all eight mice, followed by improvements in the behavioural aspect and physical appearance. The mice seemed more active as their movements were less restricted and occasional grooming was observed, compared to the previous week.

**Figure 2:**
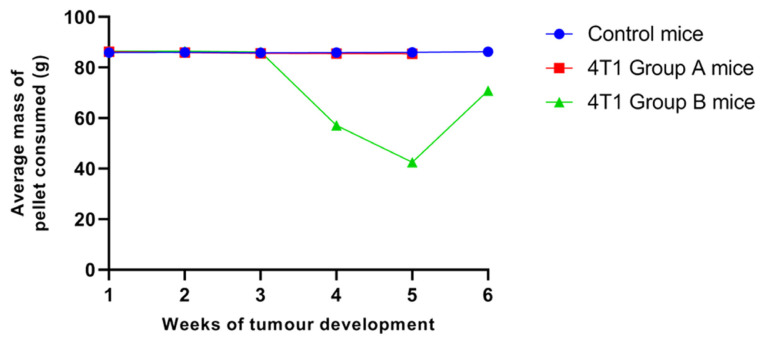
The average mass of pellet consumed by control, **(A)** and **(B)** mice groups

Despite the reduction in the size of the tumour noted in group B at week 5, the mass of pellet consumed was the lowest in that particular week as compared to the other weeks of tumour development (Figure 2). In addition, mild signs of breathing difficulties were still observed among group B mice in week 5 even though there was an improvement in the overall activities. In week 6 (day 36–42), the size of the tumour was increased when observed grossly, accompanied by severe physical changes such as the complete absence of mobility, changes in the fur pattern along with the presence of necrosis spots on the superficial skin. It was decided that the mice would be sacrificed and the duration of tumour development would not be prolonged further as to abide by the ethical policy.

### The Difference of 4T1 Tumour Volume Recorded within 4T1 Tumour Bearing Mice Groups

**Figure 3:**
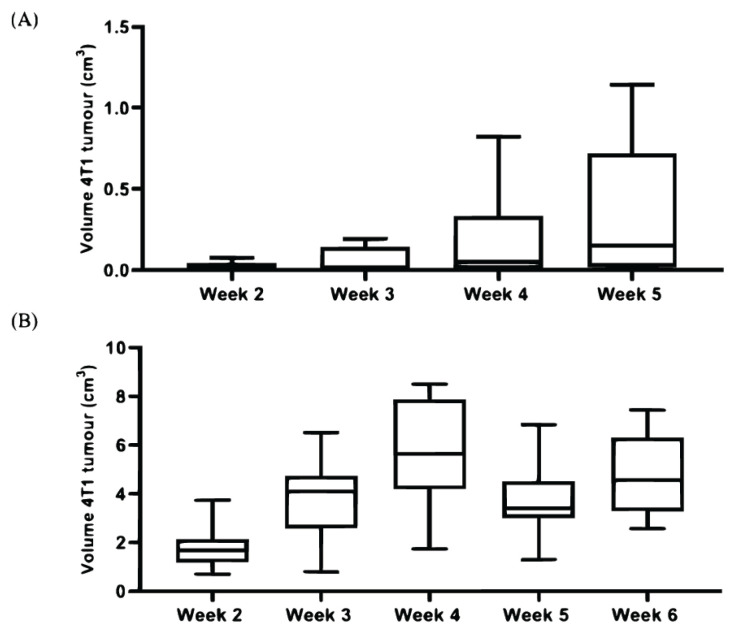
The pattern of 4T1 tumour development in A and B mice groups. **(A)** The pattern of 4T1 tumour development across different weeks in group A. **(B)** The pattern of 4T1 tumour development across different weeks in group B. Friedman analysis was conducted separately for both groups Note: The error bars represent the IQR and data are expressed in the median

Friedman analysis is a repeated-measure test that is used to portray experimental variability. Based on the Friedman analysis (Figure 3), there were significant differences in the experimental variability of the tumour volume throughout the duration of development in both groups, A (*P* = 0.0005) and B (*P* = 0.0018). In group A, the tumour development seemed stagnant from week 2–4 and in week 5, a drastic increase was observed. In group B, the experimental variability is slightly lower and exhibits a biphasic pattern. The post-hoc test revealed none of the pairs within groups A (Table 1A) and B (Table 1B) were significant.

### The Difference in the 4T1 Tumour Volume Recorded between 4T1 Tumour Bearing Mice

**Figure 4:**
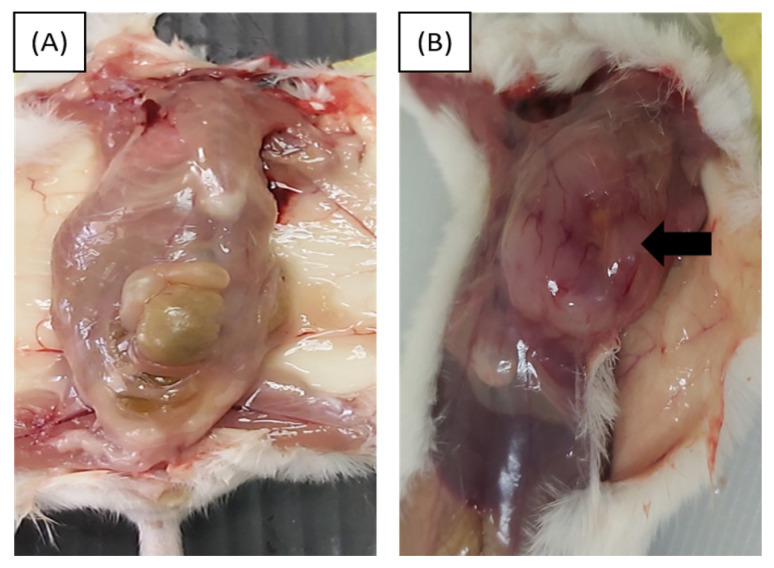
The gross examination of control and 4T1 orthotopic mice at the end of week 6. **(A)** The dissection of control mice revealed no presence of a 4T1 tumour at the 3rd mammary fat pad (week 6). **(B)** The dissection of 4T1 orthotopic mice revealed the presence of a 4T1 tumour at the 3rd mammary fat pad (week 6)

The illustration of the primary tumour in 4T1 orthotopic mice in comparison with control mice was depicted in Figure 4. Weekly comparison in the volume of the 4T1 tumour between groups A and B was conducted (Table 2). Based on the observation made, there were significant differences in the volume of 4T1 tumour between groups A and B in all five weeks (week 2–5).

### The Difference in the Net Volume of 4T1 Tumour between 4T1 Tumour-Bearing Mice Groups

**Figure 5:**
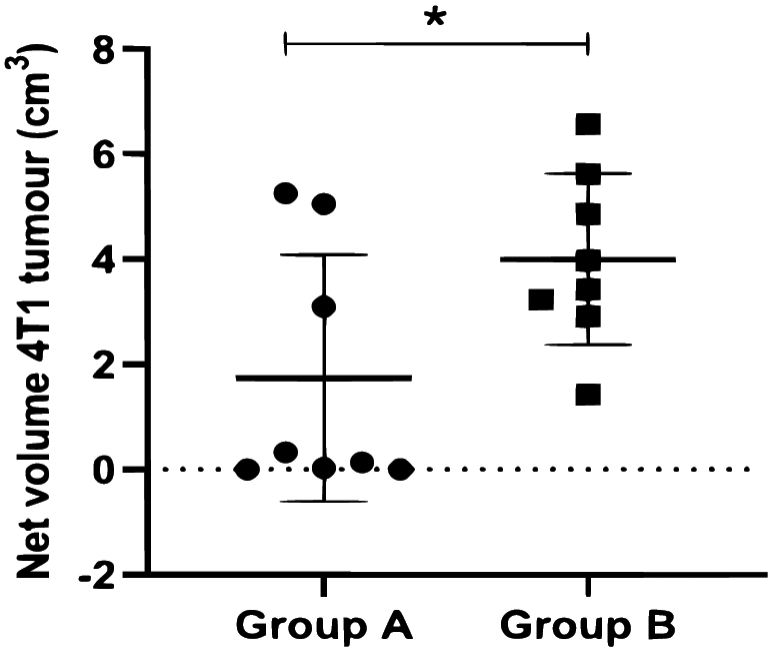
The difference in the net 4T1 tumour volume of A and B mice groups Note: The error bars represent the SD

Following organ resection, the difference in the net volume of the 4T1 tumour was found to be statistically significant between both groups A and B (*P* = 0.0420) (Figure 5), using an independent *t*-test. The mean (SD) of net 4T1 tumour volume for group A was 1.74 (2.35) and for group B, it was higher, 4.01 (1.63). The co-efficient of variation in the net volume of 4T1 tumour retrieved from group A was 135.30%. However, the variation in the net volume was only 40.79% in group B. The differences in the variation were prominent based on the dot-plot presented (Figure 5).

### Histopathological Analysis of Target Organs

**Figure 6:**
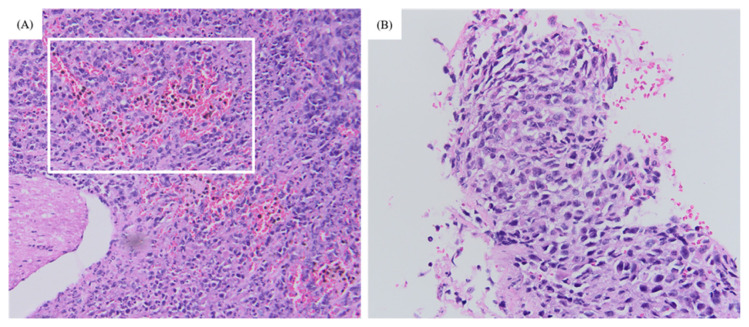
The histology of the 4T1 tumour. **(A)** The infiltration of leukocytes (within the white frame) under 20× magnification. **(B)** The presence of spindle-shaped tumour cells within the 4T1 tumour under 40× magnification

**Figure 7:**
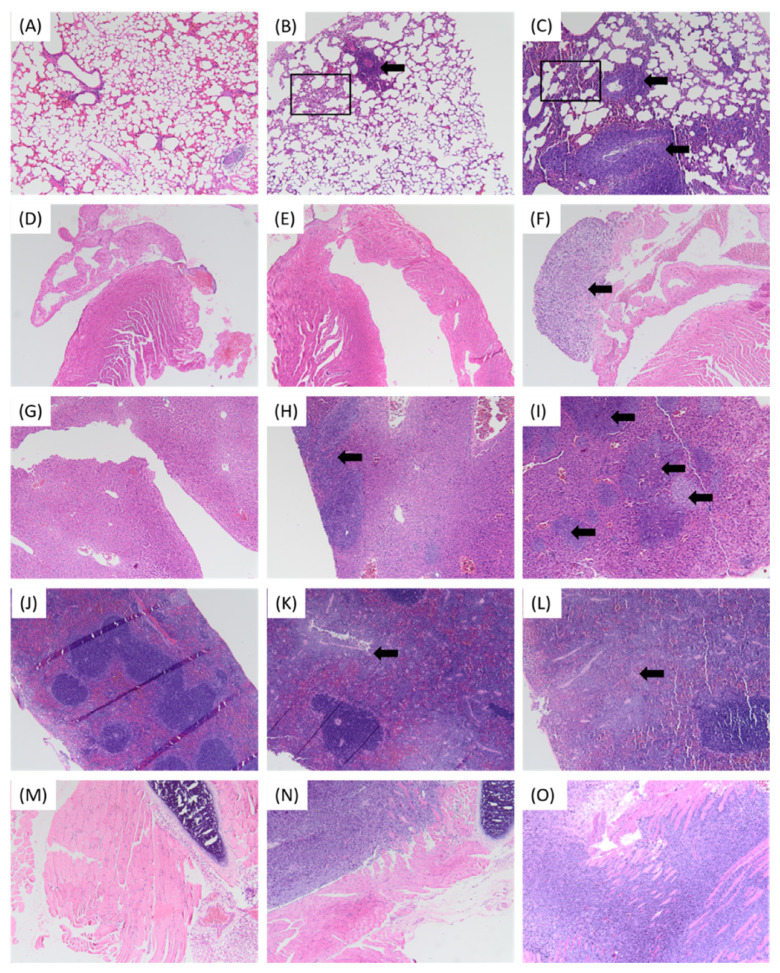
Histopathological analysis of target organs. **(A)** The normal histology of lungs in the control mice. **(B)** The histology of lungs from group A mice was slightly different from those of control mice. Occasionally, there was the presence of a cluster of tumour cells (marked by the black arrow) followed by the thickening of the alveolar walls around the cluster (highlighted in the insert box). **(C)** The histology of lungs from group B was completely different in terms of the architecture of the walls which was thicker (highlighted in the insert box), followed by the presence of infiltration of cancer cells (in clusters) which could be perceived as severe (marked by the black arrows). **(D)** The normal histology of heart in the control mice. **(E)** The histology of the heart of group A mice was similar to those of control mice. **(F)** Unlike in group A and control mice, there was the presence of invasion of tumour cells (spindle-shaped) on the heart resected from group B mice (marked by the black arrow), which was similar to those seen in the 4T1 tumour. **(G)** The normal histology of liver in control mice. **(H)** The histology of the liver of group A mice was slightly different from those of control mice, where a mild invasion of tumour cells (marked by the black arrow) was observed at the edges of the lobes. **(I)** The histology of the liver from group B showed extensive signs of metastasis of tumour cells progressing from the edges to the mid-section of the lobes, in the form of multiple clusters (marked by the black arrows). **(J)** The normal histology of spleen in control mice. **(K)** The histology of the spleen of group A mice differed from those of control mice, where there was an invasion of tumour cells (marked by the black arrow). **(L)** Invasion was also seen in the histology of the spleen of group B (marked by the black arrow). However, the invasion of the tumour cells was much extensive accompanied by a vast amount of giant tumour cells. **(M)** The normal histology of rib cage in control mice. **(N)** and **(O)** Invasion of tumour cells were seen on the rib cages from groups A and B. As anticipated, the presence of spindle-shaped tumour cells matched those found in the heart which was retrieved from group B

The histology of the 4T1 tumour included the presence of spindle-shaped tumour cells and infiltration of leukocytes (Figure 6). The histology of organs retrieved from groups A and B were compared to those of control mice. Metastasis was completely absent in the lungs, heart, spleen and liver of control mice. In group A, metastasis was only seen in the lungs, liver and spleen whereas in group B, metastasis was present in all four of the target organs (Figure 7).

## Discussion

The 4T1 cells share similar molecular features as those noted in the triple-negative subtype of human breast cancer (13), which makes the 4T1 orthotopic mice an ideal model for investigations pertaining to breast cancer metastasis. The current paper was intended to evaluate the effect of passage number on the volume of tumour, the duration of tumour growth that could be endured by the host, the pattern of tumour growth, the variation in the net volume of 4T1 tumour and the extent of metastasis within the hosts.

Invasion and metastasis are generally influenced by various expressions of genes (14–15) and the regulation of biological proteins (16–17). Previous studies have reported the role of passage number in the progression of invasion and metastasis. A study by Hamad, Kqueen and Hashim (18), reported that the difference in the passage number affects the formation of invadopodia. Invadopodias are specialised actin-rich protrusions recruited by cancer cells to perform an invasion into the surroundings (19). Hamad, Kqueen and Hashim (18) reported that the formation of invadopodia in MDA-MB-231 cells is influenced by the passage number of the cells. The study added that the lower passage number (P7) exhibited a higher percentage of cells that formed invadopodia as compared to the cells of higher passage number (P35). The paper concluded that cells of lower passage number (less than P15) should be an ideal choice in studies related to the formation of invadopodia (18). This statement implies that cells with higher passage number could miss their signaling and molecule components that are responsible for the formation of invadopodia formation, in order to carry out invasion.

Besides Hamad, Kqueen and Hashim (18), several other studies have also associated passage number with the invasion and metastatic capacity of cells. A study by Urtreger et al. (20) highlighted that the local invasive nature as well as the spontaneous metastatic capacity of LM3 cells inoculated subcutaneously increased with the passage number and were associated with progressive loss of fibronectin. This was in agreement with the study reported by Welch, Neri and Nicolson (21), whereby it was stated that the metastatic potential of each tumour cell clone drifts with passage number. In 2021, a study by Cao et al. (22) highlighted the influence of increased cell passage number on the proliferation and migration capacity of HT29 cells.

In the present study, it was noted that the introduction of P9 cells reduces the capacity of the host to bear the invasiveness of cancer cells, which is reflected by the restriction in the duration of tumour development (only 30 days). The drastic increase resulted in a significant variability in the tumour volume of group A. The sudden increase in the tumour volume at week 5 may have affected the durability of the host to bear the fast-growing tumour. However, the scenario was majorly contributed only by two mice which bore the largest tumour volume in group A, thus providing little evidence on the lethal effect of P9 cells. Since a lower passage number of cells has higher chances of forming invadopodia as discussed previously, we assume that the invasiveness of P9 might be responsible for the signs of early death of 4T1 orthotopic mice and may not have distal metastatic potential. This is because metastasis was only observed in the lungs, liver and spleen of 4T1 orthotopic mice of group A. Sotillo et al. (23) highlighted that the possibilities of melanomas in Cdk4 R24C mice, exhibiting potential invasion to the neighbouring tissues but may not have distal metastatic potential. The study added that, alternatively, the early death of mice, is likely to be caused by the concomitant development of sarcomas and carcinomas, which may have prevented the distal progression of the invasive melanoma cells. However, in the present study, concomitant tumours were not seen among the 4T1 mice models, but we could agree with the fact that P9 might not possess the characteristics required to ensure the presence of effective distal metastasis.

Based on the histopathological analysis, only group B (subjected to P10) showed signs of metastasis to all the target organs. However, the severity of metastasis on the target organs did not reduce the duration of tumour development. The tumour-bearing mice of group B even reported improved overall health by week 5 and slightly reduced variability in the tumour volume throughout the 6 weeks of tumour development, unlike in group A. During week 5, mice of group A showed signs of dying as described earlier. Despite the fact that, group B exhibited significantly higher 4T1 tumour volume, the overall health of mice in group B was better compared to group A.

The greater extent of metastasis exhibited by P10 is in agreement with the findings reported by De Meulenaere et al. (24). The study mentioned that the metastatic tumour burden (both number and volume) significantly increased with the increasing passage. A study by Muff et al. (25), reported that metastasis-relevant genes were differentially expressed in late versus early passages in three cell lines of osteosarcoma. The study reported that the difference in the passage number also contributed to the aberration in the chromosome, changes in the chromosome localisation and gene dysregulation (25). This input could also resonate with the difference in the extent of metastasis between group A (P9) and group B (P10).

Based on the data generated from group A, it was evident that P9 contributes to a larger variation in the net volume of 4T1 tumour among BALB/c mice, as compared to P10. By using a higher passage number, the variation was reduced by 94.51%. In addition, the 4T1 tumours resected from group B (P10) exhibited a significantly larger mean of 4T1 tumour net volume with a lower standard deviation in comparison to those of group A (P9). Thus, compelling us to believe that the influence of passage number on the chromosomal stability or gene expression could play a role in the differences in the biological aspects of the development of 4T1 tumour and the absence of palpable tumour in some of the experimental BALB/c mice.

In the present study, a higher passage number (P10) has clearly shown to have provided a sort of stability in terms of the capacity of the mice to resist the invasiveness of the 4T1 cells up to a certain extent and thus, allowing the progression of distal metastasis. The experiment conducted using P10, was observed to have prevented early death among the 4T1 orthotopic mice and was able to prolong the duration of 4T1 tumour growth up to week 6 (42 days). In a study by Tao et al. (26), it was stated that the progression of tumour growth lasted until week 6, which was similar to those of group B (P10). However, it must be noted that the site of tumour induction was different. In the current study, we chose to grow the 4T1 tumour at the 3rd mammary fat pad (at the thoracic region) whereas the study conducted by Tao et al. (26) highlighted the growth of tumour at the 4th and 9th mammary fat pads.

The pattern of tumour growth displayed by group A (P9) over 30 days varied tremendously as compared to those of group B (P10) over 42 days. Group A displayed a stagnant development in the mean volume of the 4T1 tumour from weeks 2–5 whereas group B exhibited biphasic pattern of tumour growth with a drop in the 4T1 tumour volume in week 5. Our findings in the pattern of tumour development exhibited by group B are in agreement with those reported by Tao et al. (26). The study stated that the regression in the pattern of tumour growth in BALB/c mice signified the involvement of the immune system as such scenario did not take place in athymic nude or SCID BALB/c mice. Tao et al. (26) also added that the biphasic growth was associated with necrosis and the infiltration of leukocytes which resonates with the histology of 4T1 tumour described earlier (Figure 6). A study by DeRose et al. (27) reported that growth rates of a primary tumour of grafted breast cancer in mice were observed to increase with a serial passage, regardless of the subtypes which corresponds with the increase in the tumour growth across the period of 42 days displayed by group B (P10).

The non-significant differences in the pattern of 4T1 tumour growth observed in group A (P9) clearly showed that the progress of tumour development was not ideal and rather, it can be described as stagnant. However, the significant differences were reported in the weekly volume of 4T1 tumour among mice of group B (P10). The fluctuation and significant differences in the 4T1 tumour volume within different weeks validate the ongoing physiological progress within the host against the growing tumour.

Furthermore, tumour development did not occur at all in some of the mice in group A indicating an early rejection which is in contrast with the findings from group B. In group B, all eight mice bore 4T1 tumour at the end of the tumour development. According to an analysis by Schrörs et al. (13), 4T1 shares a similar expression of MHC class I with some of the normal tissues of the parental BALB/c mice (host) and does not exhibit MHC class II molecules which diminishes the possibilities of 4T1 cells being rejected by the immune system of the host as BALB/c is an immunocompetent mouse.

Judging by the pattern of the tumour development in groups A and B, the presentation of 4T1 antigens (immunogenicity) to the immune system could differ among passages. The relapse in the tumour development of group B, could be a result of immune evasion (28) and may be due to the involvement of myeloid-derived suppressor cells (MDSCs) which attenuates the immune responses in order to allow the survival of the tumour (26).

The attempt to highlight the role of passage number in the formation of in vivo 4T1 tumours was the paper’s greatest strength, given no previous research had been done in this area. This will assist future experiments involving the 4T1 mice model and reduce the cost required for optimisation. In terms of limitations, we believe that metastasis-related gene expression analysis should be added to further our understanding of the reasons behind the differences in tumour volume and metastatic pattern within one passage number difference. Moreover, due to the absence of gene expression analysis, we were not able to postulate as to why mice subjected to P9 showed early signs of dying as compared to P10. In future, we wish to look at the influence of other passage numbers, apart from P9 and P10, with the assistance of gene expression analysis.

## Conclusion

In a nutshell, P10 is a more ideal option for growing 4T1 tumour in BALB/c mice as compared to P9.

- The use of P10 prolongs the duration of tumour growth that could be endured by the host;
- P10 showed a biphasic pattern of the 4T1 tumour growth;
- the use of P10 reduced the variation in the net volume of the 4T1 tumour among the experimental BALB/c mice; and
- the use of P10 to develop 4T1 orthotopic mice showed better prospects in the extent of metastasis within the hosts.

## Figures and Tables

**Table 1A:** Wilcoxon test with Bonferroni`s correction was conducted as the post-hoc test for Group A

Pairs	*P*-value	Corrected *P*-value
Week 2 versus Week 3	0.063^ns^	0.375^ns^
Week 2 versus Week 4	0.031*	0.188^ns^
Week 2 versus Week 5	0.031*	0.188^ns^
Week 3 versus Week 4	0.031*	0.188^ns^
Week 3 versus Week 5	0.031*	0.188^ns^
Week 4 versus Week 5	0.031*	0.188^ns^

Notes:

ns = not significant;

* *P* < 0.05

**Table 1B:** Wilcoxon test with Bonferroni`s correction was conducted as the post-hoc test for Group B

Pairs	*P*-value	Corrected *P*-value
Week 2 versus Week 3	0.039*	0.391^ns^
Week 2 versus Week 4	0.016*	0.156^ns^
Week 2 versus Week 5	0.016*	0.156^ns^
Week 2 versus Week 6	0.008**	0.078^ns^
Week 3 versus Week 4	0.008**	0.078^ns^
Week 3 versus Week 5	0.742^ns^	7.422^ns^
Week 3 versus Week 6	0.313^ns^	3.125^ns^
Week 4 versus Week 5	0.016*	0.156^ns^
Week 4 versus Week 6	0.258^ns^	2.578^ns^
Week 5 versus Week 6	0.078^ns^	0.781^ns^

Notes:

ns = not significant;

* *P* < 0.05;

** *P* ≤ 0.01

**Table 2:** The comparison in the measurement of 4T1 tumour according to weeks of its development between group A and B

Groups	Group A	Group B	*P-*value
Week 2	0.0190 ± 0.0286	1.7770 ± 0.9285	a0.0002***
Week 3	0.0531 ± 0.0828	3.7950 ± 1.7150	b< 0.0001****
Week 4	0.1893 ± 0.2863	5.6400 ± 2.2560	c0.0002***
Week 5	0.3505 ± 0.4209	3.7000 ± 1.5960	d< 0.0001****
Week 6	–	4.7700 ± 1.7230	–

Note: Data are expressed as mean ± SD.

a,c Mann-Whitney test was conducted;

b,d Independent T-test was conducted
